# Nitrogen Deposition and Leaching from Two Forested Catchments in Southwest China — Preliminary Data and Research Needs

**DOI:** 10.1100/tsw.2001.314

**Published:** 2001-11-15

**Authors:** T. Larssen, J. Mulder, Y. Wang, X. Chen, J. Xiao, D. Zhao

**Affiliations:** Norwegian Institute for Water Research, Oslo, Norway; Department of Soil and Water Sciences, Norwegian Agricultural University, 1432 Aas, Norway; Chinese Academy of Forestry, 100091, Beijing, China; Guizhou Research Institute of Environmental Protection Science, 550002 Guiyang, China; Chongqing Institute of Environmental Science and Monitoring, 400020 Chongqing, China

**Keywords:** nitrogen deposition, nitrogen saturation, China, forest, nitrate leaching

## Abstract

Increased nitrogen deposition has resulted in increased nitrogen pools and nitrogen leaching in European and North American forest soils. The development in Asia in general, and China in particular, suggests increased deposition of reduced nitrogen from changes in agricultural practices and of oxidized nitrogen from rapid growth of the transportation sector. Decreased nitrogen retention in forested areas in the future may cause increased NO_3_^–^ leaching and, thus, acidification and eutrophication in surface waters. The differences in climate, ecosystems, land use, and deposition history make direct application of knowledge from studies in Europe and North America difficult. In Southwest China the potential for nitrogen mobilization from forest soils may be high because of the warm and humid climate, resulting in high decomposition rates of soil organic matter. However, there are very few data available for quantifying the suspected potential for increased nitrogen leaching in forest ecosystems. Here we present data from two forested catchments, dominated by Masson pine (Pinus massoniana), near Guiyang and Chongqing, respectively, in Southwest China. The present nitrogen deposition is moderate, estimated in the range from 10 to 40 kg N ha^–1^ year^–1^. The C/N ratios of the soils are generally below 15. Nitrate concentrations in soil water are rather variable in space, with highest values of several hundred microequivalents per liter. The turnover rate of nitrogen in the forest ecosystem is quite high compared to the atmospheric deposition rate. At present, nitrate runoff from the catchments is low and intermediate in Guiyang and Chongqing, respectively. More research is needed to improve our ability to predict future nitrogen leaching from subtropical Asian coniferous forests.

## INTRODUCTION

Until recently, the concerns regarding long-range transported air pollutants and their effects in China have focused on sulfur. Official plans, as well as independent scientific assessments, suggest that stabilization of the sulfur emissions in China is possible[1,2]. Nitrogen emissions, on the other hand, are likely to increase substantially, and impacts of nitrogen emissions to the atmosphere will become of increasing importance in Asia in general, and China in particular[3,4,5,6]. There are several possible environmental consequences of increased nitrogen emissions, including formation of tropospheric ozone, acidification, and eutrophication of terrestrial and aquatic ecosystems. Increased nitrogen deposition to forest ecosystems can lead to nitrogen leaching from forested watersheds. Most studies related to nitrogen deposition and forest ecosystems have been conducted in temperate regions in Europe and North America. However, as such systems typically are nitrogen limited, nitrogen retention in the catchment is expected to cause a considerable time delay (of several decades) in nitrate leaching. Recently, it has been suggested that the C/N ratio of organic soil horizons may be used as an indicator for nitrogen saturation; in temperate coniferous forest catchments, C/N ratios of 15–25 seem to be typical threshold values for nitrate leaching[7]. Low C/N ratios are common in tropical and subtropical soils[8], but there are no systematic compilations of soil C/N ratios, N deposition, and N leaching for tropical forest soils. Nitrate leaching from several forest catchments has been studied in Japan, where a clear seasonal trend with high nitrate in runoff is seen during the wet and warm summer[9]. In China, the alkalinity of surface water is commonly high[10]; hence, high $NO_3^-$ export to streams does not necessarily cause substantial surface water acidification[9] and acidification of soil and soil water may be more important.

There are important differences between ecosystems in the southern part of China and the temperate regions of Europe and North America. It is therefore important to evaluate how knowledge from research in temperate regions can be transferred to Chinese systems. In this paper we present preliminary results related to the deposition and turnover of nitrogen in two small, forested catchments in Southwest China already affected by high sulfur deposition. The sites were originally established for studies of acidification from sulfur. However, the collected data are also relevant for studies of effects from increased nitrogen deposition. In addition to the two catchments, we have soil data for seven additional forested sites in southern China. On the basis of these data, we discuss possible future impacts of increased nitrogen deposition and point out future research needs.

## SITE DESCRIPTIONS AND DATA

The two forested sites lie near the cities Guiyang and Chongqing in Southwest China (Fig. 1). The Liu Chong Guan (LCG) site is located about 10 km from Guiyang and the Tie Shan Ping (TSP) site about 25 km from the Chongqing urban area. Acid deposition research has been going on for several years, and chemical data are available for 3 years for wet and throughfall deposition, runoff water, soils, and soil water. The available data from LCG are from the period August 1992 to April 1995. The catchment as well as data quality have been presented and discussed elsewhere[11]. From the TSP site, the data were collected from March 1995 to March 1998[12]. Both sites receive high levels of atmospheric sulfur pollution (8 to 12 g S $m^{-2}$ $year^{-1}$). The sites are dominated by Masson pine (*Pinus massoniana*) forest, planted in the early 1960s after deforestation during the Chinese “great leap forward” in 1958. The TSP site has a more homogenous pine forest, while LCG contains more broadleaf trees. The soils at both sites are acidic yellow soils on sandstone bedrock, typical for the Sichuan and Guizhou provinces. The LCG site is located at an altitude of about 1350 m; the TSP site at about 450 m. The annual precipitation is relatively similar at the two sites (1175 mm at LCG and 1100 mm at TSP), while TSP has higher

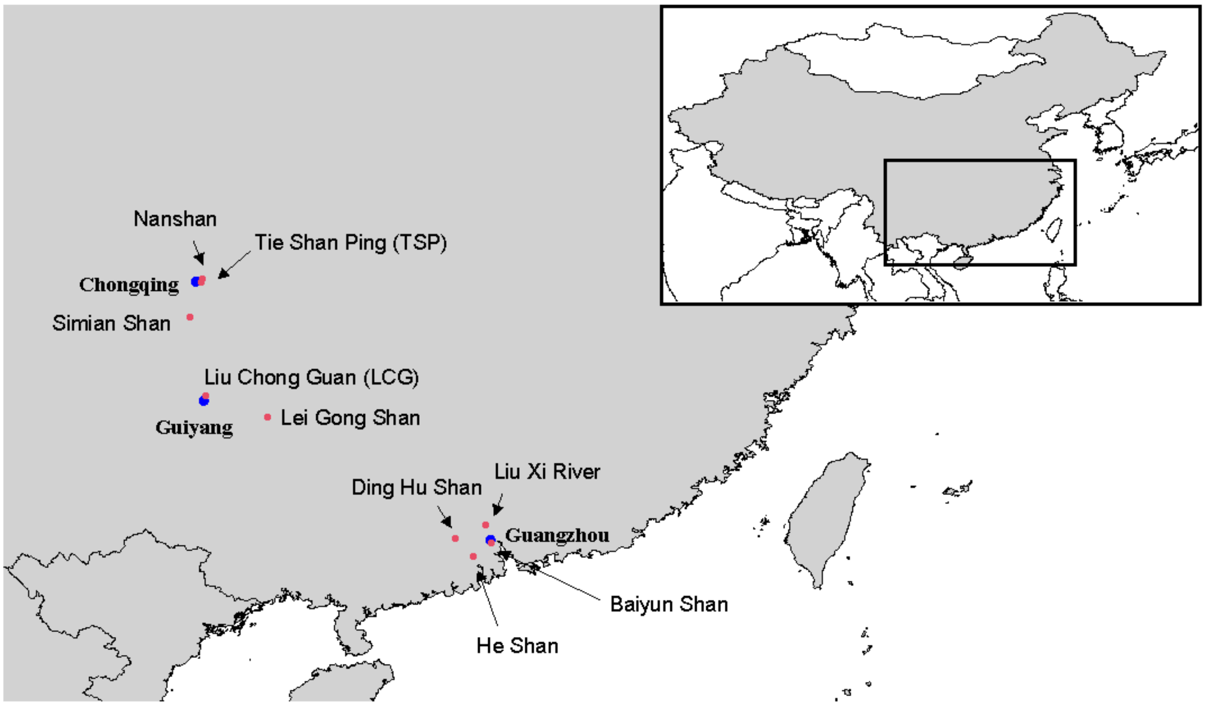

**FIGURE 1.** Map showing the location of the sampling sites.

temperatures (the warmest monthly average is 29°C at TSP and 24°C at LCG; the coldest monthly average is 8°C at TSP and 5°C at LCG). The bulk of rain falls from April through October.

At the LCG site, *in situ* net mineralization of nitrogen was studied at seven different plots during a 60-day period from April through May 1999, using 15-cm-long PVC tubes with a diameter of 4.6 cm (10 replicates per plot). The content of $NH_4^+$ and $NO_3^-$ was determined per individual soil horizon using 2M KCl. The length of the individual soil horizons in each of the 15-cm-long cores was recorded. The initial content of $NH_4^+$ and $NO_3^-$ in the soil horizons was determined using a separate set of 10 undisturbed soil cores per plot at the start of the in situ incubation experiment in early April.

## RESULTS

The two sites are quite different in terms of nitrogen deposition, as LCG receives considerably lower deposition of both $NH_4^+$ and $NO_3^-$ compared to TSP (Table 1). The median value for total annual nitrogen input at LCG was 1.9 kg N ha$^{-1}$ using bulk deposition and 5 kg N ha$^{-1}$ using throughfall. The corresponding figures for TSP were 15 kg N ha$^{-1}$ for bulk deposition and 38 kg N ha$^{-1}$ using throughfall (Table 1). As both sites are located relatively close to cities, the dry deposition must be expected to contribute significantly, although this has not been quantified. The higher nitrate deposition at TSP is likely related to more heavy pollution from transportation sources in Chongqing, as compared to LCG and Guiyang. The large difference between the two sites regarding ammonium deposition is uncertain, as there are no obvious differences in the extent of agricultural activities in the areas where the sites are located. There is a considerable seasonal variation in the deposition concentrations, with highest concentrations in the winter due to low amounts of precipitation. The variation in monthly fluxes is relatively small.

The differences observed in deposition from the two catchments are reflected in the nitrogen concentration in the runoff water, with low concentrations in LCG and higher concentrations in TSP (Table 1; Fig. 2). At LCG, nitrate contributes to the total anionic charge to a very small extent and sulfate dominates, with more than 95% of the anionic charge. At TSP, sulfate is less dominant and nitrate typically contributes 5 to 20% of the total anionic charge. No clear seasonal patterns are seen in the data, although this may be due partly to the large variance in the data. This is different from observations in North America and Europe, where clear depression in $NO_3^-$ concentrations is seen during spring and summer (growing season) and higher concentrations in the dormant season[13]. In Japan, on the other hand, the highest concentrations were seen in summer[9], which in Japan, as in Southwest China, is the wet season.

As for stream water, $NO_3^-$ concentrations in soil water are generally highest at TSP (Fig. 3). At TSP, $NO_3^-$ concentrations of 200 to 400 µeq l$^{-1}$ were common at all sampling locations. At LCG, some locations had very low $NO_3^-$ concentrations (below detection limit), while others had very high concentrations (medians above 400 µeq l$^{-1}$).

At both LCG and TSP, the C/N ratios in the soil decrease downwards from >15 in the upper soil layer to <10 in the deeper layer (Table 2). The nitrogen storage in the upper 60 cm of the soil was estimated from analyses of total nitrogen in soil samples (Table 2) and analyses of soil density. The total nitrogen storage was estimated to 7400 kg ha$^{-1}$ at LCG and 5100 kg ha$^{-1}$ at TSP. This is in the same order of magnitude as found in North American and European sites in the Integrated Forest Study[14].

**TABLE 1**
**Annual Nitrogen Deposition and Runoff and Range of Monthly Nitrogen Concentration in Deposition and Runoff at Sites LCG and TSP**

| | TSP $NO_3^-$ | TSP $NH_4^+$ | TSP Total N | LCG $NO_3^-$ | LCG $NH_4^+$ | LCG Total N |
|---|---|---|---|---|---|---|
| **Deposition:** | | | | | | |
| Median annual input (10th–90th percentiles), kgN ha$^{-1}$ | | | | | | |
| Precipitation (bulk): | 4 (2–7) | 10 (6–16) | 14 (8–23) | 0.9 (0.7–1.0) | 0.9 (0.5–8.0) | 1.8 (1.2–9) |
| Throughfall: | 9 (3–22) | 29 (10–57) | 38 (13–79) | 3.5 (1.6–13) | 1.5 (0.3–17) | 5 (1.9–30) |
| Range, monthly median concentration, µeq l$^{-1}$ | | | | | | |
| Precipitation (bulk): | 9–132 | 17–150 | | 2–32 | 1–16 | |
| Throughfall: | 13–514 | 125–878 | | 7–129 | 0–39 | |
| **Runoff:** | | | | | | |
| Median annual output (10th–90th percentiles), kg N ha$^{-1}$ | 6 (3–12) | 0.8 (0.2–2) | 6.8 (3.2–14) | 0.5 (0.3–1.5) | 0.3 (0.2–0.6) | 0.8 (0.5–2.1) |
| Range, monthly median stream concentration, µeq l$^{-1}$ | 23–103 | 5–22 | | 3–13 | 2–8 | |

*Note:* The data are calculated by taking medians and percentiles for each month, and for all 2 to 3 years (from 0 to 11 samples were taken per month per year; no year had data for all months). The resulting concentrations were used together with the monthly precipitation norms to estimate fluxes.

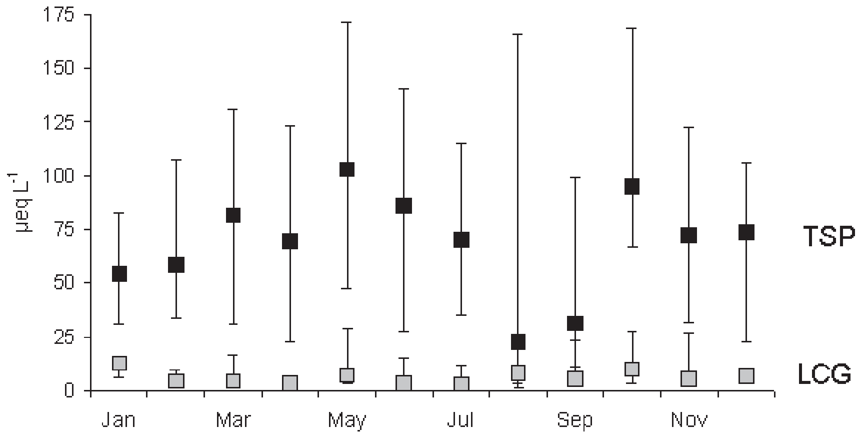

**FIGURE 2.** Monthly concentrations of $NO_3^-$ in runoff. The data shown are medians and 10th and 90th percentiles based on the available samples for each month for all years. Three sampling locations for each site are included. The sampling frequency was weekly, but fewer samples were taken in the winter due to low flow.

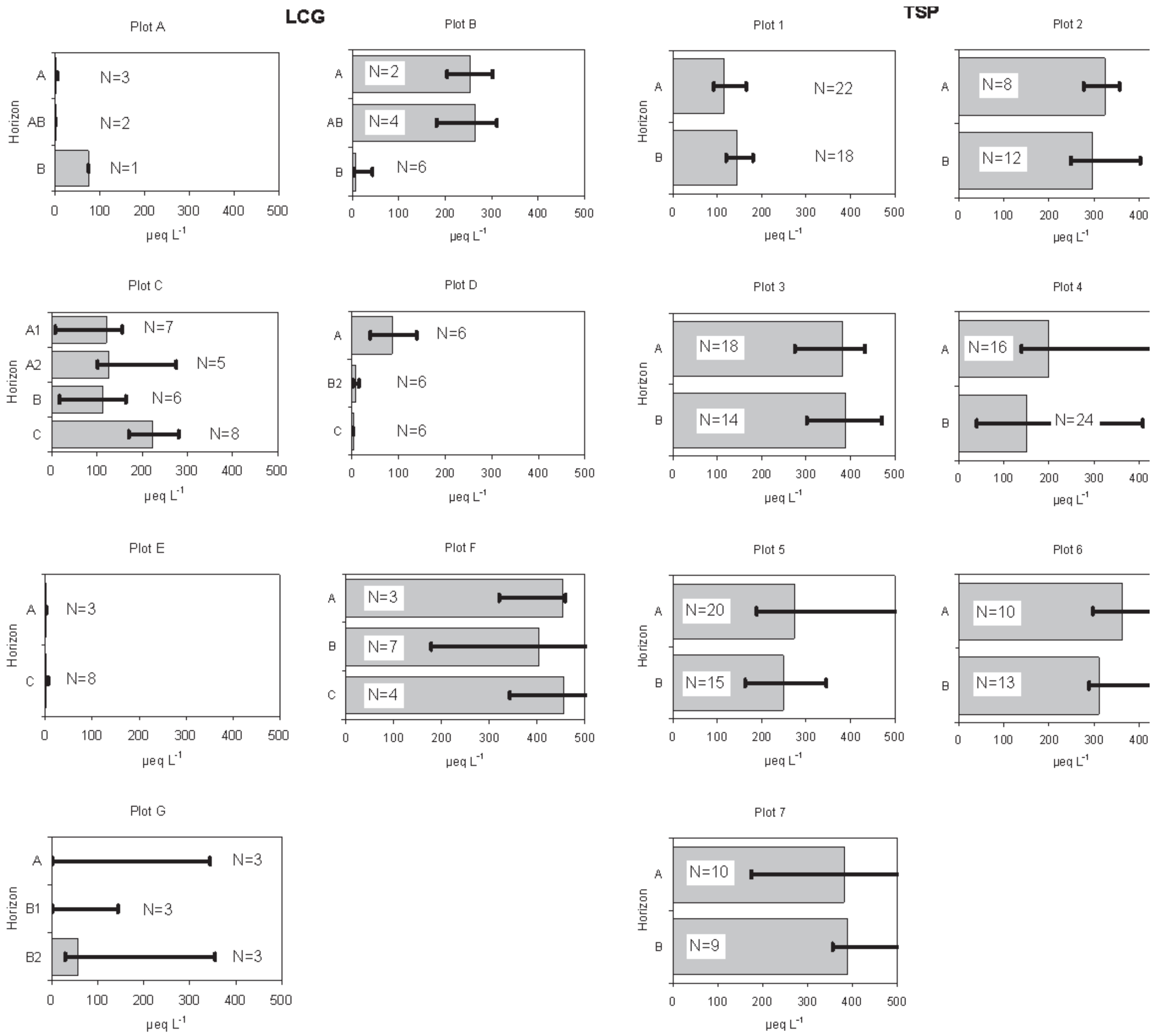

**FIGURE 3.** Median, and 25th and 75th percentiles of $NO_3^-$ concentrations in the soil water samples from LCG (left part) and TSP (right part). Results for different plots in the catchment and different soil horizons (lysimeters) are shown. Calculation of medians and quartiles are based on data for all years; number of samples (N) is shown.

**TABLE 2**
**Total Carbon and Nitrogen Content and C/N Ratio in LCG and TSP**

| Horizon | N | Tot. C (%) | Tot. N (%) | C/N |
|---|---|---|---|---|
| **LCG** | | | | |
| H | 5 | 30.3 (8.8) | 1.7 (0.5) | 17.5 (1.0) |
| A | 11 | 5.7 (3.1) | 0.4 (0.2) | 15.4 (2.4) |
| AB | 8 | 2.8 (2.1) | 0.2 (0.1) | 12.4 (1.9) |
| B | 11 | 0.9 (0.4) | 0.1 (0.02) | 9.0 (2.3) |
| **TSP** | | | | |
| A | 1 | 7.44 | 0.46 | 16.1 |
| B1 | 1 | 1.35 | 0.12 | 11.0 |
| B2 | 1 | 0.74 | 0.09 | 8.1 |

*Note:* LCG samples are available from a range of plots and the given values are averages (with standard deviations in parathesis) for the catchment. TSP data are from one plot only.

Nitrogen mineralization was measured *in situ* in LCG. The average net ammonification in the upper 15 cm of the soil was 25 kg ha$^{-1}$ (standard deviation 7 kg ha$^{-1}$) for the 60-day period. Net nitrification amounted to 3 kg ha$^{-1}$ (standard deviation 2 kg ha$^{-1}$) for the same period. Assuming constant rates year round, this would imply net average nitrogen mineralization rates of 160 kg ha$^{-1}$ year$^{-1}$. This is high compared to values reported for temperate forests[15] and may indicate higher turnover rates in subtropical systems. However, our extrapolation may represent an overestimation, because the winter season is considerably colder than the study period of April to May[16]. Nevertheless, the annual atmospheric deposition rate of N at LCG is low compared to the rate of the internal cycling. Additional research (at TSP and other sites) is needed to investigate the effects of increased atmospheric deposition rates on the internal cycling and leaching of $NO_3^-$.

## DISCUSSION

The nitrogen deposition at the investigated sites can be characterized as low to moderate at present, but is expected to increase rapidly in the future. Nitrate concentrations in surface water at LCG are very low; nitrate is significant in runoff at TSP, although sulfate is by far the dominant of the strong acid anions. Nitrate leaching is significant at TSP and the annual nitrate flux in the streams is from 20 to 50% of the nitrogen measured in deposition. This may indicate that these ecosystems leach more nitrogen when the input increases. In Japan, high nitrogen concentrations were observed in runoff water in the summer season[9], which is different from the typical picture in Europe and North America. This was explained by high mineralization and nitrification rates due to the warm and wet summer[9]. A similar seasonal pattern has been observed in southern China[17], but cannot be observed in our study due to noise in the data. The partly high nitrate concentrations observed in soil solution suggest a high nitrogen turnover rate, which is also in agreement with the high nitrogen mineralization rates found at LCG. The high soil-solution nitrate concentration at some plots may also indicate that nitrogen fixation can be important. In a Chinese survey, it was reported that on average 20 to 25% of the soil nitrogen was fixed $NH_4^+$ in the subtropical region[18].

The C/N ratios for the soils at LCG and TSP were quite similar, in the range of 6–18. In Fig. 4 we compare with data from seven additional sites in Southwest and South China (Fig. 1). The sites were divided into four groups according to climatic conditions and vegetation types. The LCG and TSP sites were, together with one additional site, grouped as central coniferous sites. These sites had similar C/N ratios as the two sites grouped as mountainous, unpolluted, coniferous sites. The sites with broadleaf vegetation in the south, on the other hand, had in general somewhat lower C/N ratios, while the one southern coniferous site showed a large span in C/N ratios with the soil depth. The C/N ratios decrease to the south, where the climate is warmer and the nitrogen turnover is expected to be higher. The soil C/N ratios for all sites are low compared to values from temperate regions, where most studies of nitrogen cycling have been carried out. Studies of tropical ecosystems show that tropical soils may have different N-dynamics, as the organic matter turnover rate is high and, hence, the nitrogen cycling is faster and more open compared to temperate regions[19,20]. Temperate systems are often N-limited, while tropical systems are commonly limited by other nutrients, such as phosphorus or base cations rather than nitrogen[19]. The forested areas in southern China may be somewhere between the temperate and the tropical in terms of N-dynamics. An important question regarding impacts of increased nitrogen deposition in China is to what extent the systems are nitrogen limited and how much increase in nitrogen is needed before most nitrogen input is leached. Results from TSP indicate that only moderate nitrogen deposition is needed before nitrogen leaching starts. However, it must also be pointed out

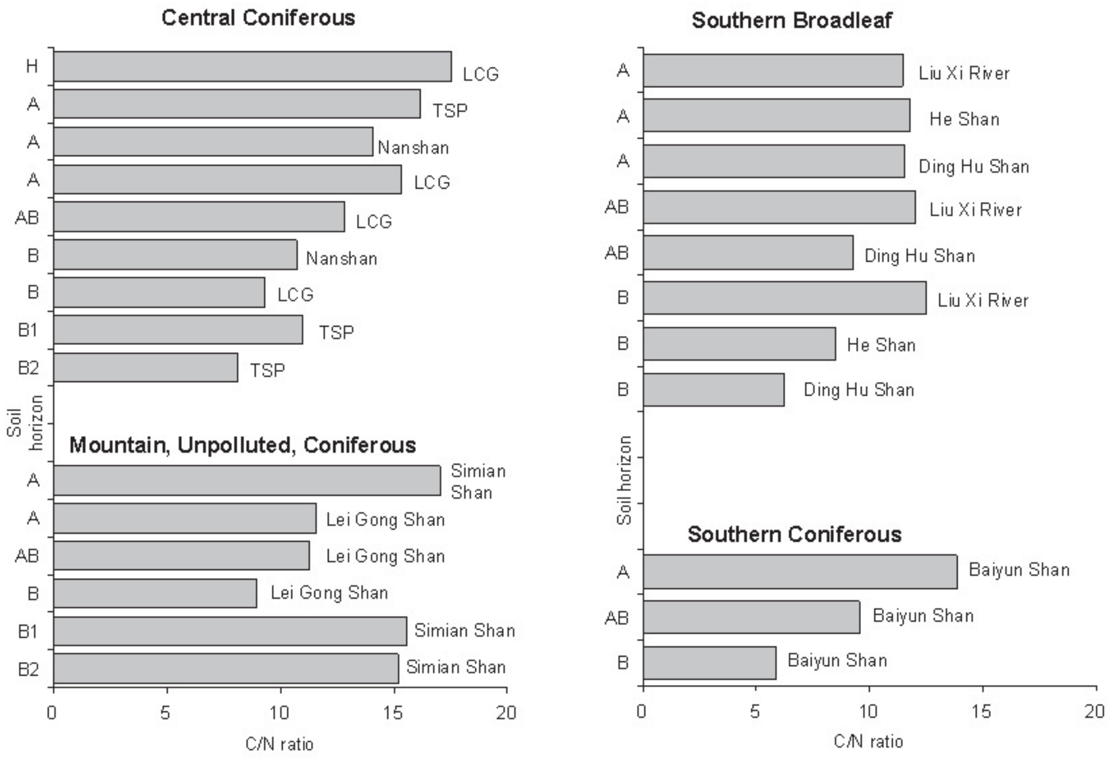

**FIGURE 4.** C/N ratios in soils at LCG, TSP, and seven other forested sites. See Fig. 1 for site locations. The sites are grouped according to geography (and hence climate) and vegetation type. Samples for different soil horizons are shown.

that the higher nitrogen deposition is one, but not the only possible, reason why the nitrogen leaching at TSP is larger than at LCG. Differences in the vegetation, that could be either different species or different disturbances, may cause higher nitrate leaching[21]. In order to understand this more fully, additional research is needed regarding the vegetation compartment of the vegetation cycling.

As very little data are available for calculation of nitrogen budgets, or about the nitrogen dynamics in China today, there is an urgent need for more research in this field. In order to be able to predict the fate of nitrogen emitted to the atmosphere in the future, better understanding of the nitrogen processes is necessary. More catchment studies with reliable data are necessary, preferably covering ranges of temperature and N-deposition. Improved quality control and data assurance are important to allow calculation of detailed budgets and to understand the seasonal dynamics of the nitrogen cycle. The N-mineralization study presented here points to the high mineralization rates, but more such studies, in different regimes of temperature, moisture, and N-deposition, would increase the usefulness of such data. The contribution from N-fixation should also be evaluated, although good quantification is difficult.

## CONCLUSIONS

The nitrogen deposition at the two investigated sites is low to moderate. At the TSP site, outside Chongqing, the nitrate concentration in the runoff water is significant and corresponds to 20 to 50% of the nitrogen deposition. At the LCG site, outside Guiyang, nitrate in runoff is very low. The C/N ratios in forest soils at the investigated sites are low compared to common ratios in soil in temperate regions. The C/N ratios in the southernmost sites appear to have lower C/N ratios.

Our data indicate that only moderate nitrogen deposition is needed before nitrate starts to leach in these ecosystems. There is a need to further improve the knowledge of when the nitrogen retention capacity is reached for Chinese forests in order to understand possible future impacts of increased nitrogen deposition in China.

| | | |
|---|---|---|
| Received: | July | 13, 2001 |
| Revised: | October | 8, 2001 |
| Accepted: | October | 15, 2001 |
| Published: | November | 15, 2001 |
